# Development of Genetically Stable *Escherichia coli* Strains for Poly(3-Hydroxypropionate) Production

**DOI:** 10.1371/journal.pone.0097845

**Published:** 2014-05-16

**Authors:** Yongqiang Gao, Changshui Liu, Yamei Ding, Chao Sun, Rubing Zhang, Mo Xian, Guang Zhao

**Affiliations:** 1 CAS Key Laboratory of Biobased Materials, Qingdao Institute of Bioenergy and Bioprocess Technology, Chinese Academy of Sciences, Qingdao, China; 2 University of Chinese Academy of Sciences, Beijing, China; 3 Institute of Oceanology, Chinese Academy of Sciences, Qingdao, China; 4 Collaborative Innovation Center for Marine Biomass Fibers, Materials and Textiles of Shandong Province, Qingdao, China; Louisiana State University and A & M College, United States of America

## Abstract

Poly(3-hydroxypropionate) (P3HP) is a biodegradable and biocompatible thermoplastic. In our previous study, a pathway for P3HP production was constructed in recombinant *Esecherichia coli.* Seven exogenous genes in P3HP synthesis pathway were carried by two plasmid vectors. However, the P3HP production was severely suppressed by strain instability due to plasmid loss. In this paper, two strategies, chromosomal gene integration and plasmid addiction system (PAS) based on amino acid anabolism, were applied to construct a genetically stable strain. Finally, a combination of those two methods resulted in the best results. The resultant strain carried a portion of P3HP synthesis genes on chromosome and the others on plasmid, and also brought a tyrosine-auxotrophy based PAS. In aerobic fed-batch fermentation, this strain produced 25.7 g/L P3HP from glycerol, about 2.5-time higher than the previous strain with two plasmids. To the best of our knowledge, this is the highest P3HP production from inexpensive carbon sources.

## Introduction

*Escherichia coli* strains are widely used as hosts for microbial production of valuable compounds, like biofuels, chemicals, polymers, and proteins, and the production processes often depend on expression of heterologous genes carried by plasmid vectors [1]. Plasmids have been regarded as important tools for microbial genetic modifications. However, plasmids are separate genetic elements and autonomously replicated, and the redundant DNA carried by plasmids may cause metabolic burden in host strains, which could result in plasmid loss [2]–[4]. For plasmid maintenance, the cloning and expression vectors harbor antibiotic resistance genes and require the addition of antibiotics to the medium. Though it is feasible at the laboratory scale, the use of antibiotics at industrial scale will increase the production cost and raise the ecological issues. Furthermore, plasmid loss can even occur with presence of antibiotics during cultivation [5].

Plasmid addiction system (PAS) is an efficient strategy to prevent the survival of plasmid-free cells due to selective killing. Up to now, three major groups of PAS have been described: (1) toxin/antitoxin-based systems, (2) metabolism-based systems, and (3) operator repressor titration systems [1]. PASs have been successfully used in metabolically engineered strains to increase the product yield by stabilizing the plasmid in the cells, which carries the genes associated with product synthesis pathway and an addiction system. For instance, an antibiotic-free plasmid selection system based on glycine auxotrophy was constructed and used for overproduction of recombinant protein [6]. To maintenance the plasmid carrying cyanophycin synthesis gene *cphA*, Kroll et al. [7] established a novel anabolism-based addiction system. This system consisted of two components: an *E. coli ispH* mutant that cannot synthesize isopentenyl pyrophosphate (IPP), an essential precursor for isoprenoid biosynthesis, and a synthetic plasmid harboring *cphA* gene and the relevant genes of a foreign IPP-producing mevalonate pathway. The resultant strain revealed a plasmid stability of 100% and improved cyanophycin production.

Chromosomal gene integration (CGI) is another strategy to stabilize foreign genes. For example, pyruvate decarboxylase gene *pdc* and alcohol dehydrogenase II gene *adhB* from *Zymomonas mobilis* were integrated into the *E. coli* chromosome for ethanol biosynthesis, and the integration improved the stability of the *Z. mobilis* genes in *E. coli* and ethanol production [8]. Recently, a method was developed to insert multiple desired genes into target loci on *E. coli* chromosome and up to six copies of *lacZ* gene were simultaneously integrated into different loci. The β-galactosidase activity increased corresponding to the copy number of inserted *lacZ* genes [9]. To a certain extent, multiple insertions have resolved the main problem of CGI, low expression level of recombinant protein due to a low copy number.

**Figure 1:**
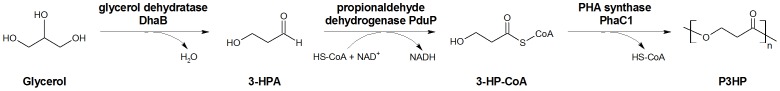
P3HP synthesis pathway used in this study. 3-HPA, 3-hydroxypropionaldehyde; 3-HP-CoA, 3-hydroxypropionyl coenzyme A.

Poly(3-hydroxypropionate) (P3HP) is a biodegradable and biocompatible plastic exhibiting high rigidity, ductility, and exceptional tensile strength in drawn films, and was regarded as one of the alternatives to petrochemical-derived plastic [10]. The biosynthesis of P3HP and 3HP-containing copolymers was previously dependent on structurally related precursors, such as 3HP, acrylate and 1,3-propanediol [11]–[14]. However the addition of these expensive precursors increased P3HP production cost. To solve this problem, we constructed a recombinant *E. coli* strain to synthesize P3HP using inexpensive carbon source glycerol (Figure 1) [15]. The genes involved in P3HP synthesis were cloned into two plasmids: the glycerol dehydratase and its reactivating factor genes, *dhaB123* and *gdrAB*, from *Klebsiella pneumoniae* were inserted in the expression vector pACYCDuet-1 to generate plasmid pWQ04, and the propionaldehyde dehydrogenase gene *pduP* from *Salmonella typhimurium* and polyhydroxyalkanoate synthase gene *phaC1* from *Cupriavidus necator* were carried by pWQ02. Under the optimized culture conditions, the recombinant *E. coli* strain accumulated 10.1 g/L P3HP (representing 46.4% of the cell dry weight) in a fed-batch fermentation.

**Figure 2:**
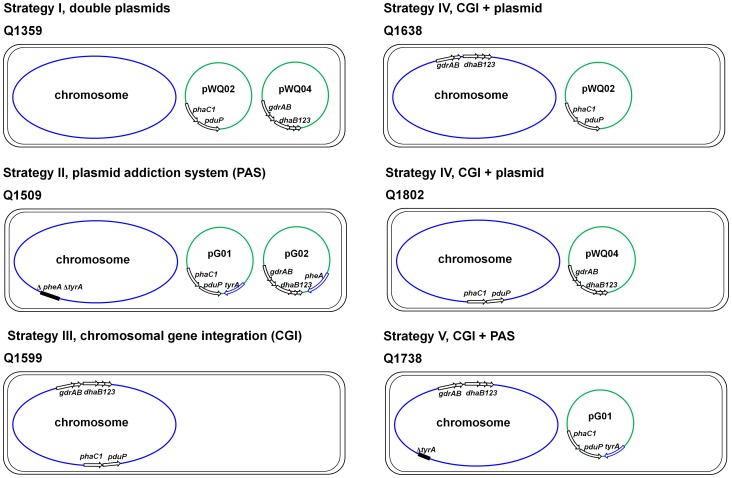
Schematic representation of the strains and strategies for P3HP production used in this study.

To optimize the P3HP production strain, 5 strategies (Figure 2) were designed and tested in this study. Strategy I used the previously constructed plasmids pWQ02 and pWQ04. Strategy II was designed to construct a phenylalanine/tyrosine-auxotrophy based PAS and all the genes associated with P3HP synthesis were integrated in *E. coli* chromosome in Strategy III. Strategy IV strains carried portion of genes involved in P3HP synthesis on chromosome and others on plasmid, which was further developed using a tyrosine-auxotrophy based PAS to improve the plasmid stability in Strategy V. As a result, the Strategy V strain Q1738 produced 25.7 g/L P3HP from glycerol in aerobic fed-batch fermentation, 2.5-time higher than the previous report.

## Materials and Methods

### Bacterial Strains and Growth Conditions

All strains and plasmids used in this study are listed in Table 1. *E. coli* DH5α was used as the host to construct and store all recombinant plasmids, *E. coli* χ7213 strain was used for preparation of all suicide vectors, and *E. coli* BL21(DE3) strain was used for protein expression and P3HP production. Bacteria were grown at 37°C in Luria-Bertani (LB) broth unless specified. Diaminopimelic acid (DAP) (50 µg/ml) was used for the growth of χ7213 strain. When necessary, antibiotics were added at final concentration of 100 µg/ml for ampicillin and 34 µg/ml for chloramphenicol. LB agar containing 10% sucrose was used for *sacB* gene-based counter selection in allelic exchange experiments.

**Table 1:** Bacteria strains and plasmids used in this study.

Strains or plasmid	Description	Source
***E. coli*** ** strains**		
DH5α	F^–^ *supE*44 Δ*lacU*169 (*φ*80 *lacZ* Δ*M15*) *hsdR*17 *recA*1 *endA1 gyrA*96 *thi*-1 *relA*1	lab collection
BL21(DE3)	F^–^ *ompT gal dcm lon hsdSB* (rB^−^ mB^−^) λ(DE3),	lab collection
?7213	*thi-1 thr-1 leuB6 glnV44 fhuA21 lacY1 recA1 RP4-2-Tc*::Mu λ*pir* Δ*asdA4 Δzhf-2*::Tn10	[29]
Q1475	Δ*pheA* Δ*tyrA*	BL21(DE3)
Q1463	Δ*prpR*::*lacI* P_T7_ *gdrAB* P_T7_ *dhaB123*	BL21(DE3)
Q1599	Δ*prpR*::*lacI* P_T7_ *gdrAB* P_T7_ *dhaB123* Δ*mtlA*::P_T7_ *phaC pduP*	BL21(DE3)
Q1633	Δ*prpR*::*lacI* P_T7_ *gdrAB* P_T7_ *dhaB123* Δ*ascF*::P_T7_ *phaC pduP* Δ*mtlA*::P_T7_ *phaC pduP*	BL21(DE3)
Q1693	Δ*prpR*::*lacI* P_T7_ *gdrAB* P_T7_ *dhaB123* Δ*ascF*::P_T7_ *phaC pduP* Δ*mtlA*::P_T7_ *phaC pduP* Δ*ebgR*::P_T7_ *phaC pduP*	BL21(DE3)
Q1736	Δ*prpR*::*lacI* P_T7_ *gdrAB* P_T7_ *dhaB123* Δ*ascF*::P_T7_ *phaC pduP* Δ*mtlA*::P_T7_ *phaC pduP* Δ*ebgR*::P_T7_ *phaC pduP* Δ*melR*::P_T7_ *phaC pduP*	BL21(DE3)
Q1779	Δ*mtlA*::P_T7_ *phaC pduP*	BL21(DE3)
Q1734	Δ*tyrA* Δ*prpR*::*lacI* P_T7_ *gdrAB* P_T7_ *dhaB123*	BL21(DE3)
Q1359	BL21(DE3) carrying pWQ02 and pWQ04	[15]
Q1509	Q1475 carrying pG01 and pG02	BL21(DE3)
Q1638	Q1463 carrying pWQ02	BL21(DE3)
Q1802	Q1779 carrying pWQ04	BL21(DE3)
Q1738	Q1734 carrying pG01	BL21(DE3)
**Recombinant plasmids**		
pWQ02	rep_pBR322_ Amp^R^ *lacI* P_T7_ *phaC pduP*	[15]
pWQ04	rep_p15A_ Cm^R^ *lacI* P_T7_ *gdrAB* P_T7_ *dhaB123*	[15]
pG01	rep_pBR322_ Amp^R^ *lacI* P_T7_ *phaC pduP* TT P*_lac1-6_ tyrA*	pWQ02
pG02	rep_p15A_ Cm^R^ *lacI* P_T7_ *gdrAB* TT P*_pheA_ pheA* P_T7_ *dhaB123*	pWQ04
**Suicide plasmids**		
pRE112	*oriT oriV sacB cat*	[16]
pG03	Δ*pheA* Δ*tyrA*	pRE112
pG04	Δ*tyrA*	pRE112
pG05	Δ*ascF*::P_T7_ *phaC pduP*	pRE112
pG06	Δ*mtlA*::P_T7_ *phaC pduP*	pRE112
pG07	Δ*ebgR*::P_T7_ *phaC pduP*	pRE112
pG08	Δ*melR*::P_T7_ *phaC pduP*	pRE112
pLC01	Δ*prpR*::*lacI* P_T7_ *gdrAB* P_T7_ *dhaB123*	pRE112

### Construction of Recombinant Plasmids

The primers used are listed in Table S1 in File S1. The plasmid pG01 was constructed using PCR fragments containing the *tyrA* coding region generated with primers 366 and 367 and BL21(DE3) chromosomal DNA as a template, which were digested with *Xho*I and then ligated with pWQ02 digested by the same enzyme. The plasmid pG02 was constructed using PCR fragments containing *pheA* coding region generated with primers 364 and 365 and BL21(DE3) chromosomal DNA as template, which were digested with *Afl*II and *Hin*dIII and then ligated with pWQ04 digested by the same enzymes.

### Construction of BL21(DE3) Strains with Chromosomal Mutations

The primers used are listed in Table S1in File S1. The mutations were constructed using suicide vector pRE112 as previously described [16]. For the *pheA tyrA* deletion, two pairs of primers, 369/370 and 371/372, were used to amplify approximately 500-bp fragments upstream and downstream of these genes from BL21(DE3) chromosome, respectively. The two fragments were then joined by PCR using primers 369 and 372. The PCR product was digested with *Sac*I and *Xba*I and then ligated between the *Sac*I and *Xba*I sites of vector pRE112 to generate plasmid pG03. The *pheA tyrA* mutation was introduced into BL21(DE3) by allelic exchange using suicide vectors pG03. A similar strategy was used to construct *tyrA* mutation using suicide vector pG04 constructed with primers 569/570 and 571/572.

To integrate the *dhaB123* and *gdrAB* genes into *prpR* locus on BL21(DE3) chromosome, two pairs of primers, 268/269 and 270/271, were used to amplify approximately 500-bp fragments upstream and downstream of *prpR* gene from BL21(DE3) chromosome, respectively. The two fragments were then joined by PCR using primers 268 and 271. The PCR product was digested with *Sac*I and *Nhe*I and then ligated between the *Sac*I and *Xba*I sites of vector pRE112 to generate plasmid pRE112-Δ*prpR*. Then the *Kpn*I-*Xba*I fragment containing *dhaB123 gdrAB* genes from plasmid pWQ04 was inserted into the corresponding sites of plasmid pRE112-Δ*prpR* to generate suicide vector pLC01, which was used to mediate the allelic exchange to generate Δ*prpR*::*lacI* P_T7_ *gdrAB* P_T7_ *dhaB123* strain. A similar strategy was used to construct suicide vectors pG05, pG06, pG07, and pG08 to generate the chromosomal gene integration of Δ*ascF*::P_T7_ *phaC pduP*, Δ*mtlA*::P_T7_ *phaC pduP*, Δ*ebgR*::P_T7_ *phaC pduP*, and Δ*melR*::P_T7_ *phaC pduP*, respectively.

### Shake Flask Cultivation

The strain was inoculated into 500 ml baffled Erlenmeyer flasks containing 100 mL of minimal medium, which contains 3 g/L glucose, 20 g/L glycerol, 1.5 g/L KH_2_PO_4_, 3 g/L (NH_4_)_2_SO_4_, 1 g/L citric acid, 1 g/L citrate sodium, 1.9 g/L KCl, 3 g/L MgSO_4_, 0.138 g/L FeSO_4_·7H_2_O, 4.5 mg/L vitamin B_1_, and 1 ml of trace element solution. The trace element solution contained (per liter): 0.37 g (NH_4_)_6_Mo_7_O_24_·4H_2_O, 2.47 g H_3_BO_4_, 1.58 g MnCl_2_·4H_2_O, 0.29 g ZnSO_4_·7H_2_O, 0.25 g CuSO_4_·5H_2_O. The culture broth was inoculated with the overnight culture and incubated in a gyratory shaker incubator at 37°C and 200 rpm. The cells were induced at OD_600_∼0.6 with 0.05 mM IPTG and further incubated at 30°C. 5 µM of vitamin B_12_ (VB_12_) and appropriate antibiotic were added every 12 h. The cell dry weight (CDW) and P3HP yield were determined after 48 h culturing. All shake flask experiments were carried out in triplicates.

### Fed-batch Fermentation

Fed-batch cultures were carried out in a Biostat B plus MO5L fermentor (Sartorius Stedim Biotech GmbH, Germany) containing 3 L of minimal medium as described above. During the fermentation process, pH was controlled at 7.0 via automated addition of 5 M KOH and antifoam 204 was used for foam control. The dissolved oxygen (DO) concentration was maintained at 5% saturation by associating with agitation from 400 rmp to 800 rpm and aeration with the airflow rate of 2 liters per min. After the initial carbon sources were nearly exhausted, fed-batch mode was commenced by feeding a solution containing 10 M glycerol at 0.5 mL/min. The expression of exogenous genes was initiated at an OD_600_ of 12 by adding 0.05 mM IPTG and 5 µM VB_12_. IPTG, VB_12_ and appropriate antibiotic were added every 12 h.

### Cell Harvest, P3HP Extraction and Characterization

Cells of *E. coli* were harvested with centrifugation at 5000×g for 15 min, and washed with distilled water twice. To determine CDW, cell pellets were lyophilized, and the CDW was gravimetrically determined. P3HP was extracted from lyophilized cells with hot chloroform in a Soxhlet apparatus, and precipitated by ice-cold ethanol as described [17]. P3HP structure was confirmed by NMR analysis using an Avance III 600 NMR spectrometer (Bruker, Switzerland) as described previously [15].

### Analysis of Antibiotic Concentration

Samples were withdrawn from the cultivation flask and centrifuged at 5000×g for 10 min. The supernatant was filtered using 0.22-µm filter, and concentrations of ampicillin and chloramphenicol were determined using Agilent 1200 HPLC System as described previously [18], [19].

### Analysis of Plasmid Stability

To determine plasmid stability, samples were withdrawn from the cultivation flask at assigned time, diluted and spread onto LB agar plates with or without antibiotics supplemented. Ampicillin and chloramphenicol were used for plasmids pWQ02/pG01 and pWQ04/pG02, respectively. The plates were incubated for 16 h at 37°C, and the colony-forming units (CFU) were determined and analyzed by comparing the CFU on LB agar plates containing antibiotics and CFU on LB agar plates without antibiotics.

#### SDS-PAGE

The strain Q1599 and *E. coli* BL21(DE3) were grown in MM and induced by 0.05 mM IPTG. The cells were harvested by centrifugation and lysed by sonication. The whole-cell lysate was used for SDS-PAGE. Protein concentration was determined using the Bradford Protein Assay Kit (Tiangen, China). Proteins were separated in 12% acrylamide gels and visualized with Coomassie brilliant blue R250.

## Results

### P3HP Production and Plasmid Stability of Strategy I Strain

In our previous study, the genes involved in P3HP synthesis pathway were carried by two plasmids pWQ02 and pWQ04 [15]. An *E. coli* BL21(DE3) strain carrying pWQ02 and pWQ04, named as Q1359, was used to test the P3HP production and plasmid stability with the presence and absence of antibiotics. Without the addition of antibiotics, strain Q1359 accumulated 0.34 g/L P3HP representing 15.5% of the CDW under shaking flask condition. When ampicillin and chloramphenicol were added into the culture media, P3HP production and content increased to 0.52 g/L and 17.3%. After 48 h of cultivation, cultures were appropriately diluted and spread onto LB agar plates with and without the antibiotics to calculate the plasmid stability. Most of the cells lost their plasmids even with antibiotic selection (Table 2), and it was assumed that the segregational plasmid instability was caused by two reasons. First, plasmid duplication increased metabolic burden of the strain. Secondly, the antibiotic in medium was degraded during culturing process. To test this speculation, the ampicillin and chloramphenicol concentration in medium was determined by HPLC. Surprisingly, no antibiotic can be detected after 48-h cultivation even with periodic antibiotic addition every 12 h, indicating that the antibiotic degraded very fast under concentrations used in this study.

**Table 2:** P3HP production and plasmid stability of plasmid-containing strains.

Strains	antibiotics	CDW (g/L)	P3HP (g/L)	P3HP content	pWQ02 or pG01 stability	pWQ04 or pG02 stability
Q1359	–	2.27±0.19	0.34±0.04	15.5±1.2%	6.7±1.0%	4.4±1.1%
	+	3.07±0.22	0.52±0.02	17.3±2.1%	12.8±2.3%	8.2±1.7%
Q1509	–	2.33±0.31	0.55±0.03	22.5±1.1%	42.9±3.7%	27.2±0.9%
	+	2.59±0.29	0.84±0.07	31.2±0.5%	59.1±2.4%	36.9±3.1%
Q1638	–	4.67±0.23	2.01±0.09	42.6±1.7%	76.4±4.0%	–
	+	4.54±0.36	2.34±0.05	52.4±4.2%	87.3±2.8%	–
Q1802	–	2.78±0.06	0.56±0.06	20.3±1.9%	–	6.5±0.5%
	+	3.32±0.14	0.83±0.05	25.0±0.9%	–	13.1±.06%
Q1738	–	4.46±0.24	1.99±0.06	45.0±2.9%	80.8±1.7%	–
	+	4.51±0.18	2.40±0.04	53.7±3.4%	90.2±4.0%	–

The experiment was performed under shake flask condition in triplicate.

### Construction and Characterization of Strain with PAS

**Figure 3:**
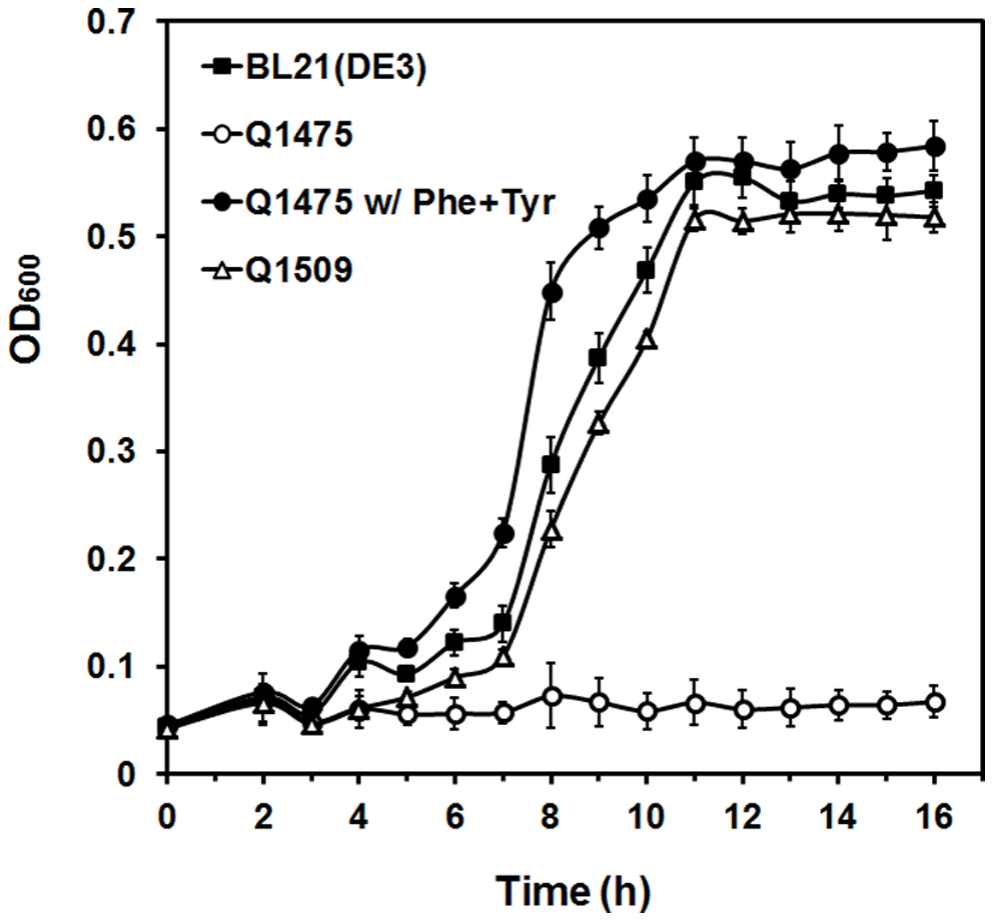
Growth of *E. coli* strains with and without the phenylalanine/tyrosine- auxotrophy based PAS, tested using minimal medium. The experiment was performed under shake flask condition in triplicate.

To improve P3HP production and plasmid stability, a phenylalanine/tyrosine-auxotrophy based PAS was designed and constructed. The biosynthetic pathways of aromatic amino acids phenylalanine and tyrosine share the first step, from chorismate to prephenate catalyzed by bifunctional chorismate mutase/prephenate dehydratase PheA or TyrA. Besides that, PheA also carries out the second step in phenylalanine synthesis, converting prephenate into 2-keto-phenylpyruvate [20], and TyrA is responsible for the formation of 4-hydroxyphenylpyruvate from prephenate in tyrosine synthesis [21]. The *pheA* and *tyrA* genes are located next to each other in *E. coli* chromosome, and transcription of these 2 loci proceeds in opposite direction. In this study, an *E. coli* Δ*pheA* Δ*tyrA* mutant Q1475 was constructed using suicide vector pRE112 [16]. The chromosomal knockout of *pheA* and *tyrA* was verified by PCR and DNA sequencing. As shown in Figure 3, strain Q1475 was not able to grow in minimal medium. When phenylalanine and tyrosine were added, growth level and rate was similar to the wild-type *E. coli* BL21(DE3) strain, confirming the Phe^−^Tyr^−^ phenotype of strain Q1475.

To complement the phenylalanine/tyrosine-auxotrophy phenotype, the *tyrA* and *pheA* genes were cloned into pWQ04 and pWQ02, and the resulting plasmids were named as pG01 and pG02, respectively (Fig. S1 in File S1). For *pheA* gene, the structure gene and its own promoter region were amplified from *E. coli* chromosomal DNA. As *tyrA* is the second gene in cluster and does not have its own promoter, a constitutive promoter P*_lac1–6_* [22] was fused to the *E. coli tyrA* coding region by PCR. In order to rule out the possible interferer between transcription of *pheA*/*tyrA* gene and P3HP synthesis associated genes, a bi-directional Rho-independent transcriptional terminator [23] was added behind the *pheA* and *tyrA* structural genes. The resulting plasmids pG01 and pG02 were confirmed by DNA sequencing, and transformed into Q1475 to generate Strategy II strain Q1509. In minimal medium without addition of phenylalanine and tyrosine, strain Q1509 revealed a similar growth with the wild-type strain (Fig. 3).

To verify P3HP accumulation and plasmid stability, strain Q1509 was cultivated as described above. The results showed that the phenylalanine/tyrosine-auxotrophy based PAS greatly increased the stabilities of pG01 and pG02 (Table 2). In absence of antibiotics, Q1509 exhibited plasmid stabilities of 42.9% for pG01 and 27.2% for pG02, more than 6-time higher than Strategy I strain Q1359. When antibiotics were added into the medium, the plasmid stabilities of pG01 and pG02 increased to 59.1% and 36.9%, respectively, about 4.5-time higher than strain Q1359. Unfortunately, the P3HP production did not raise in proportion of the plasmid stabilities. Only 0.55 g/L and 0.84 g/L P3HP were harvested under the condition without and with antibiotics addition, about 1.5-time higher compared with Q1359 (Table 2).

### Construction and Characterization of CGI Strains

To insert the P3HP synthesis associated genes into *E. coli* BL21(DE3) chromosome, a set of suicide plasmid was constructed based on the vector pRE112 [16]. For example, the flanking regions of *prpR* gene were amplified and linked up with each other by overlap extension PCR, and the restriction sites of *Kpn*I and *Xho*I were introduced at the connection point by primer design. This fragment was cloned into the vector pRE112 to generate pRE112-Δ*prpR*. Then an *Xho*I-*Kpn*I fragment from pWQ04, encoding transcriptional regulator LacI and glycerol dehydratase system, was inserted into the corresponding site of pRE112-Δ*prpR*, and the resulting plasmid was defined as pLC01, which was used to mediate the allelic exchange. After two rounds of selection based on the positive marker chloramphenicol resistance gene *cat* and negative marker levan-sucrase gene *sacB* from *Bacillus* spp. [24], we obtained the strain Q1463 carrying chromosomal copy of *dhaB123* and *gdrAB* genes (Figure S2 in File S1and Table 1). The *phaC1* and *pduP* genes were inserted into the *mtlA* locus, mediated by suicide vector pG06 similarly, to generate Strategy III strain Q1599 (Figure 2, Figure S3 in File S1 and Table 1). The *prpR* gene and *mtlA* gene encode propionate catabolism regulator and mannitol-specific phosphotransferase system (PTS) enzyme IIA component, respectively. Under conditions used in this study, the *prpR* and *mtlA* mutations shouldnot affect the cell metabolism.

Cultivated in minimal medium for 48 h, strain Q1599 accumulated 0.26 g/L P3HP, which represented 6.4% of the CDW and was much lower than the P3HP productions of Q1359 and Q1509. To figure out the reason of low P3HP production in strain Q1599, SDS-PAGE analysis of whole-cell lysate was performed. Compared with the control strain *E. coli* BL21(DE3), DhaB1 and GdrA were observed as distinct bands with the expected molecular weights on SDS-PAGE, however we cannot find the bands for PhaC1 and PduP at appropriate position. It is assumed that the low copy number limited the expression level of *phaC1* and *pduP* genes, so another three copies of these two genes were inserted into the chromosomal loci of *ascF*, *ebgR* and *melR*. These three genes are all involved in degradation of carbon compounds like cellobiose and lactose. As these disaccharide molecules are absent, the inactivation of those genes should not be detrimental to cell metabolism and growth. The result of shake flask cultivation showed that the P3HP production increased with the crescent copy number of *phaC1* and *pduP* genes (Table 3), to the similar level of strains Q1359 and Q1509. Although this strain is stable and antibiotic-free, its P3HP production was still too low.

**Table 3:** P3HP production of strains with various copy numbers of *phaC1* and *pduP* genes.

Strains	copy number of *phaC1* and *pduP* genes	CDW (g/L)	P3HP (g/L)	P3HP content
Q1599	1	4.02±0.26	0.26±0.04	6.4±0.4%
Q1633	2	3.58±0.14	0.38±0.03	10.7±0.8%
Q1693	3	3.37±0.21	0.44±0.01	13.2±1.4%
Q1736	4	2.65±0.26	0.50±0.01	18.9±0.3%

The experiment was performed under shake flask condition in triplicate.

### Combination of CGI Strategy and Plasmid Vector

To further improve the P3HP production, we constructed the Strategy IV strains, in which the CGI strategy and plasmid vector were used simultaneously. The strain Q1638 carries a chromosomal copy of genes encoding glycerol dehydratase and its reactivatase and plasmid-borne *phaC1* and *pduP* genes, while in strain Q1802 the *phaC1* and *pduP* genes were integrated into the *mtlA* locus and the *dhaB* and *gdrAB* genes were carried by plasmid pWQ04 (Figure 2).

The strains Q1638 and Q1802 were inoculated into minimal medium to test the P3HP production and plasmid stability. After 48 h of cultivation in shake flasks, the strain Q1638 accumulated 2.01 g/L and 2.34 g/L P3HP without and with addition of ampicillin, respectively, while the strain Q1802 only produced 0.56 g/L and 0.83 g/L P3HP under the same conditions (Table 2). In respect of plasmid stability, 76.4% of strain Q1638 cells still carried plasmid pWQ02 at the end of cultivation even without addition of ampicillin, however only 13.1% of strain Q1802 cells possessed the expression plasmid pWQ04 with the presence of chloramphenicol (Table 2).

Based on Strategy IV strain Q1638, we developed Strategy V strain Q1738 as following: the chromosomal *tyrA* gene was knocked out and plasmid pWQ02 was replaced by pG01 (Figure 2). In shake flask cultivation, strain Q1738 revealed similar P3HP production and slightly higher plasmid stability than strain Q1638 (Table 2). Compared with original strain Q1359, strains Q1638 and Q1738 presented about 4.5-time higher P3HP production and only required the addition of ampicillin in the growth process. Even without the usage of antibiotics, the P3HP production of strains Q1638 and Q1738 was still about 4-time higher than that of strain Q1359 with presence of ampicillin and chloramphenicol.

### Fed-batch Fermentation

**Figure 4:**
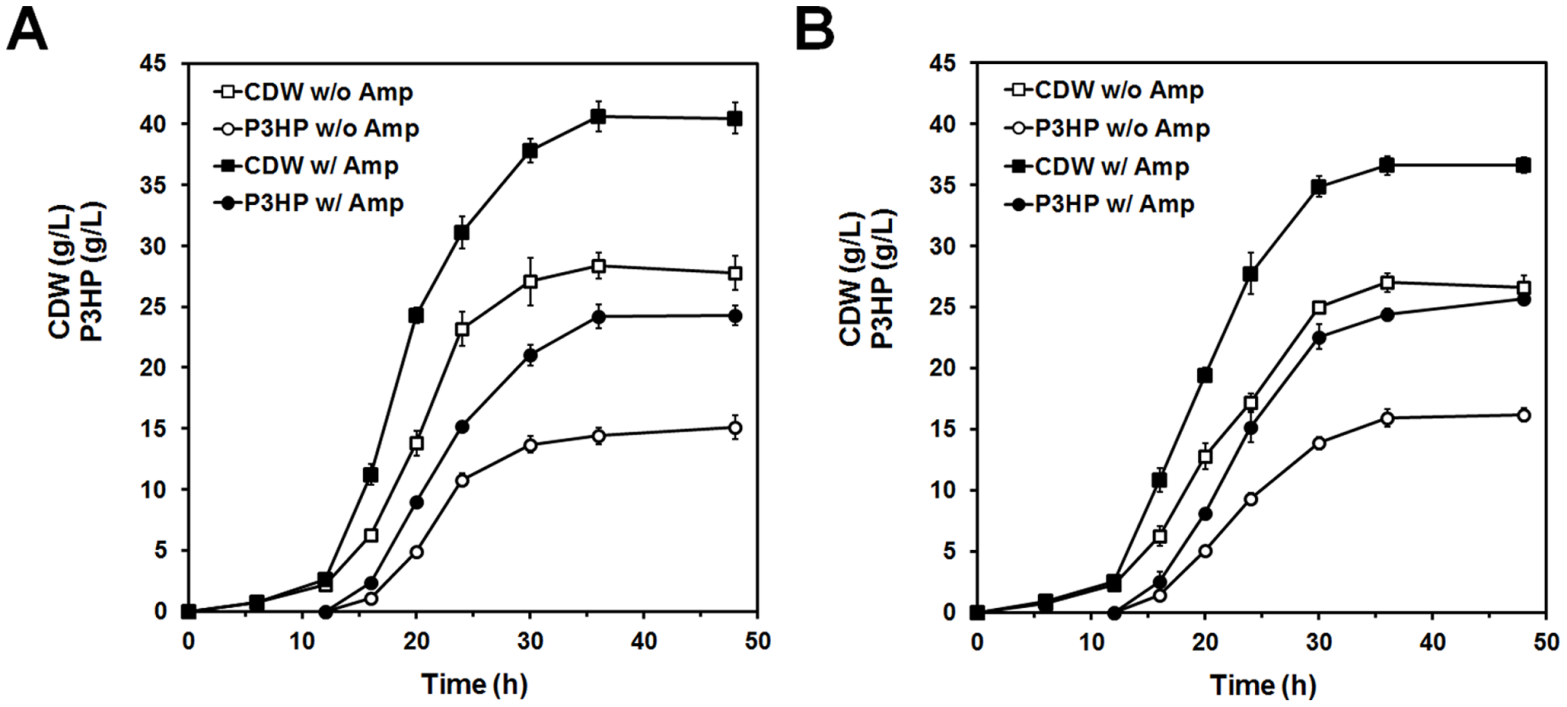
Time profiles for CDW and P3HP production during aerobic fed-batch fermentation of Strain Q1638 (A) and Q1738 (B). The experiment was performed in triplicate.

To evaluate the P3HP production in a scalable process, fed-batch fermentation of Q1638 and Q1738 was carried out at 5-L scale under aerobic condition. Cell growth and P3HP accumulation were monitored over the course of fermentation. As shown in Figure 4, CDW and P3HP reached the maximum in 36 h. With presence of ampicillin, the P3HP productions of trains Q1638 and Q1738 were 24.3 g/L (58.1% of CDW) and 25.7 g/L (67.9% of CDW), respectively. Even without antibiotic addition, 15.1 g/L and 16.2 g/L P3HP was accumulated by trains Q1638 and Q1738, respectively, higher than the previously reported P3HP yield from glycerol [15].

## Discussion

In this study, we are trying to construct a genetically stable strain for P3HP biosynthesis. As shown in Table 2, the stability of plasmid pWQ02 in stain Q1638 and plasmid pWQ04 in strain Q1802 was significantly improved when compared with strain Q1359. In strains Q1738 and Q1509, similar phenotype of plasmid pG01 was also observed. All these three strains with increased plasmid stability contain only one plasmid, whereas two in strains Q1359 and Q 1509. This phenomenon indicated that the plasmid stability would decrease if two or more types of plasmid exist in the same strain. It was reported that the segregative plasmid stability decreased with the size increasing, and the metabolic burden caused by plasmid duplication is a major reason for plasmid loss [25], [26]. Multiple plasmids brought about heavier burden on cell metabolism obviously, and a single plasmid had better stability reasonably.

Another noticeable result is that the CDW and P3HP production of strain Q1638 were much higher than that of Q1359, and the only difference between these strains is the copy number of glycerol dehydratase genes. Besides burden caused by plasmid duplication, the toxic product of glycerol dehydratase was also responsible for the low P3HP content. Glycerol dehydratase converts glycerol into 3-hydroxypropionaldehyde, which is a major component of antimicrobial substance Reuterin and inhibits the growth of some bacteria, yeasts and protozoa [27], [28]. The difference between strains Q1509 and Q1738 can be explained in the same way, and the intermediate toxicity was also the reason that the CDW and P3HP production were significantly lower when ampicillin was not supplemented in strains Q1638 and Q1738 (Figure 4). They carry a chromosomal copy of glycerol dehydratase gene, and the other genes involved in P3HP synthesis were borne by plasmids with ampicillin resistance. When ampicillin was absent, the plasmid instability increased, resulting in intracellular accumulation of 3-hydroxypropionaldehyde and growth depression.

In sum, the microbial P3HP production from glycerol was improved greatly by constructing a genetically stable *E. coli* recombinant strain. To overcome the strain instability due to plasmid loss, two strategies, amino acid anabolism based PAS and chromosomal integration, were tested. Finally, a combination of those two methods led to the best result. Our recombinant strain Q1738 produced 25.7 g/L P3HP from glycerol in aerobic fed-batch fermentation. To the best of our knowledge, this is the highest P3HP production from inexpensive carbon sources.

## Supporting Information

File S1 **This file contains Figure S1–S3 and Table S1.**
(DOCX)
