# Supplementary material for: Development of Genetically Stable Escherichia coli Strains for Poly(3-Hydroxypropionate) Production
Source: PLoS One. 2014 May 16;9(5):e97845. doi: 10.1371/journal.pone.0097845 (PMC4023983; doi:10.1371/journal.pone.0097845)
Supplement: File S1 — This file contains Figure S1–S3 and Table S1. (DOCX) [file pone.0097845.s001.docx]

Table S1 Primers used in this study

| **Primers** | **Sequences** |
| --- | --- |
| ***tyrA* cloning** | |
| 366 | ACGTCTCGAGCTTTACACTTTAAGCTTTTTATGTTTATGTTGTGTGGAACCGATGCCTTGCTGCGTG |
| 367 | ACGTCTCGAGAAAAGCCTCCGGTCGGAGGCTTTTCGGCACTGGATTATTACTGG |
| ***pheA* cloning** | |
| 364 | ATCGACTTAAGGCAGCACAAAGGCGAAGC |
| 365 | ACGTAAGCTTAAAAGCCTCCGGTCGGAGGCTTTTGATTCACATCATCCGGCACC |
| **Δ*pheA* Δ*tyrA* construction** | |
| 369 | CAGTGAGCTCCATCTCTTCCTGCGATTTCTC |
| 370 | GGCCTTATTGTTCGTCTTCG |
| 371 | CGAAGACGAACAATAAGGCCACCTCTTAAGCCACGCGAGC |
| 372 | CAGCTCTAGAACCACTGTCGGCTGGAAAGG |
| 373 | AAACGGCAGCCCTTGAGCTG |
| **Δ*tyrA* construction** | |
| 569 | CAGTGAGCTCCCATTGTTTGTTGGTCTCCG |
| 570 | GAAAGCCGCGTGTTATTGC |
| 571 | GCAATAACACGCGGCTTTCTTGATCGCGTAATGCGGTC |
| 572 | CAGCTCTAGAGACGGCAGTCTGGCAACAG |
| 573 | GTGCCATAAACGACGTTTACG |
| **Δ*prpR*::*lacI* P_T7_ *gdrAB* P_T7_ *dhaB123* construction** | |
| 268 | CAGAGCTCGGTGACCTACATCCACAAG |
| 269 | GTACCGGTACCGATCGTTCTAGACAGTTGACTGCAACAAACGGATGC |
| 270 | TCTAGAACGATCGGTACCGGTACGCTGTTTCTCGATGAG |
| 271 | ATCTGCGCTAGCGCTGCATTCGGCAGTTCAG |
| **Δ*ascF*::P_T7_ *phaC pduP* construction** | |
| 421 | CAGAGCTCCGGCGTGCTGACAAAAGG |
| 422 | GAATTGTGAGCGGATAACAATTCCCCTCTAGACATCTCGAGCCAGCAGAGGCCACAGCG |
| 423 | TTGTTATCCGCTCACAATTCCCCTATAGTGAGTCGTATTA  GGATGCACCGCGTCTTTAC |
| 424 | ATCTGCGCTAGC CGGTCGGTTGTACGCTCTGAT |
| **Δ*mtlA*::P_T7_ *phaC pduP* construction** | |
| 426 | CAGAGCTCCAAATATCGGCGCGTTTATC |
| 427 | GAATTGTGAGCGGATAACAATTCCCCTCTAGACATCTCGAGCAGCGTGAACACGCCAGTC |
| 428 | TTGTTATCCGCTCACAATTCCCCTATAGTGAGTCGTATTAGCAACTCGTCGTATGCAGG |
| 429 | ATCTGCGCTAGCCCAGACGCTCGATGACGG |
| **Δ*ebgR*::P_T7_ *phaC pduP* construction** | |
| 538 | CAGAGCTCAGCAGAACTTACCGGCTCAG |
| 539 | GAATTGTGAGCGGATAACAATTCCCCTCTAGACATCTCGAGGATACGCCAGCTTCGATTG |
| 540 | TTGTTATCCGCTCACAATTCCCCTATAGTGAGTCGTATTAAGACATTAAAAACGTCACCGG |
| 541 | ATCTGCGCTAGCTAAGGACAGAAACAGGCTGC |
| **Δ*melR*::P_T7_ *phaC pduP* construction** | |
| 532 | CAGAGCTCATGCGCCAGTATGGTCAAG |
| 533 | GAATTGTGAGCGGATAACAATTCCCCTCTAGACATCTCGAGGATGAAACAGTACATTACCGCG |
| 534 | TTGTTATCCGCTCACAATTCCCCTATAGTGAGTCGTATTATTCGGACTGTTTAATTCCTGC |
| 535 | ATCTGCGCTAGCACAGATGCGGAATGGTACGTAG |


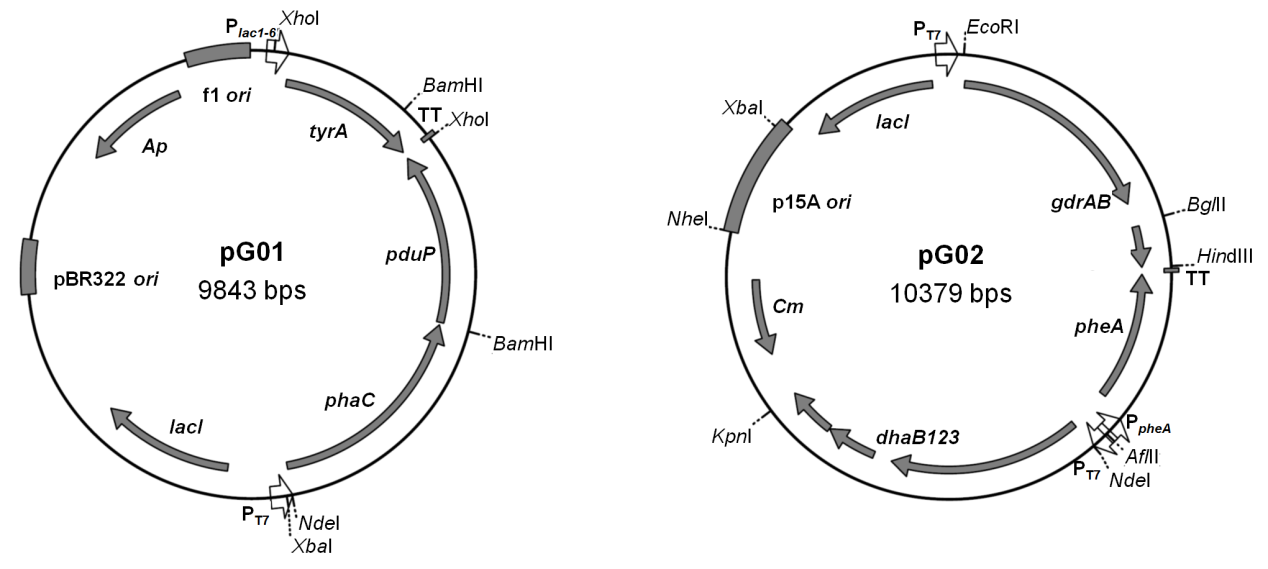


Fig. S1. The plasmids pG01 and pG02 used in this study. TT, transcriptional terminator.


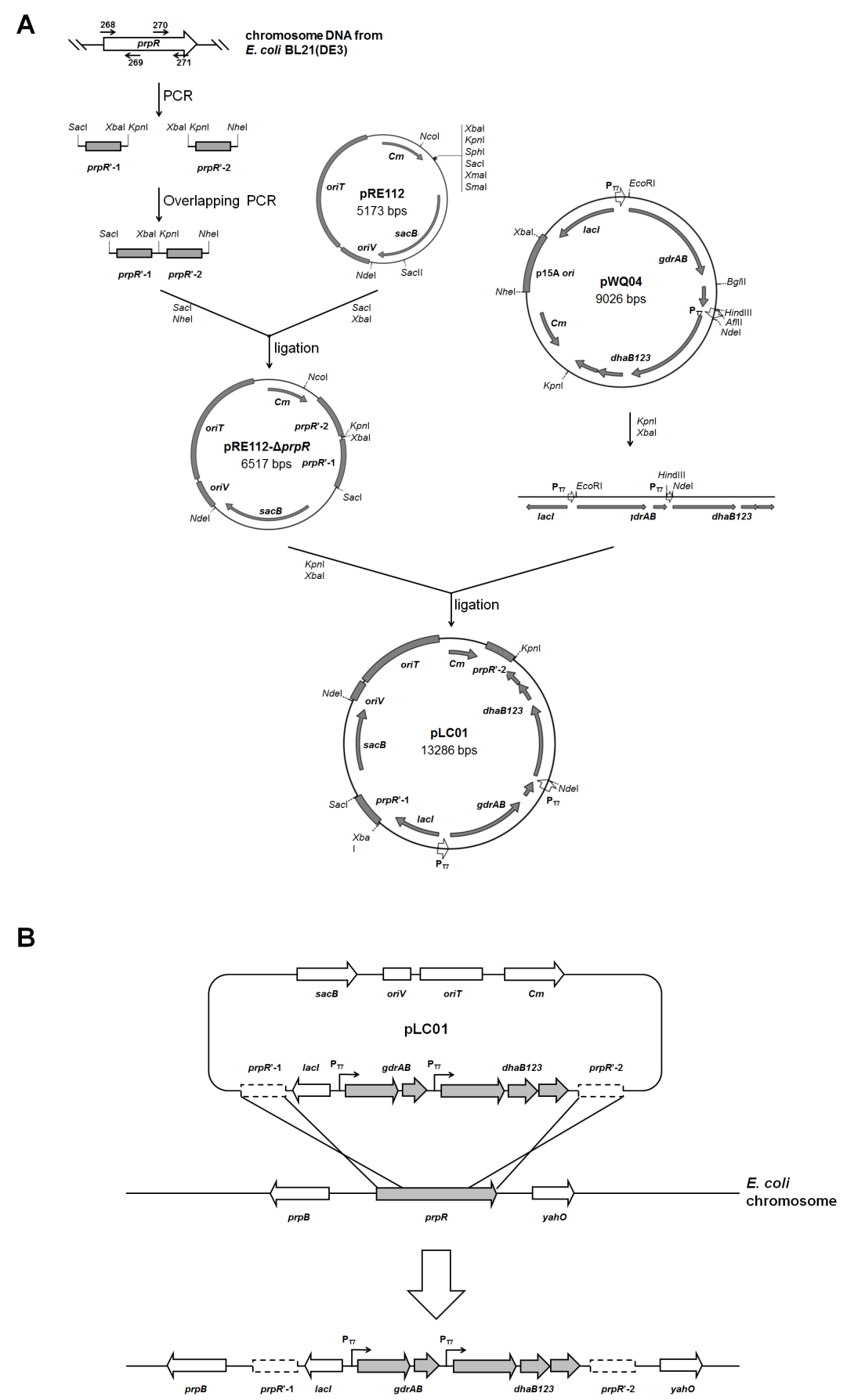


Fig. S2. Construction process of strain carrying *gdrAB* and *dhaB123* genes on chromosome. (A) Construction of suicide vector pLC01. (B) Allelic exchange between the suicide vector pLC01 and *E. coli* chromosome.


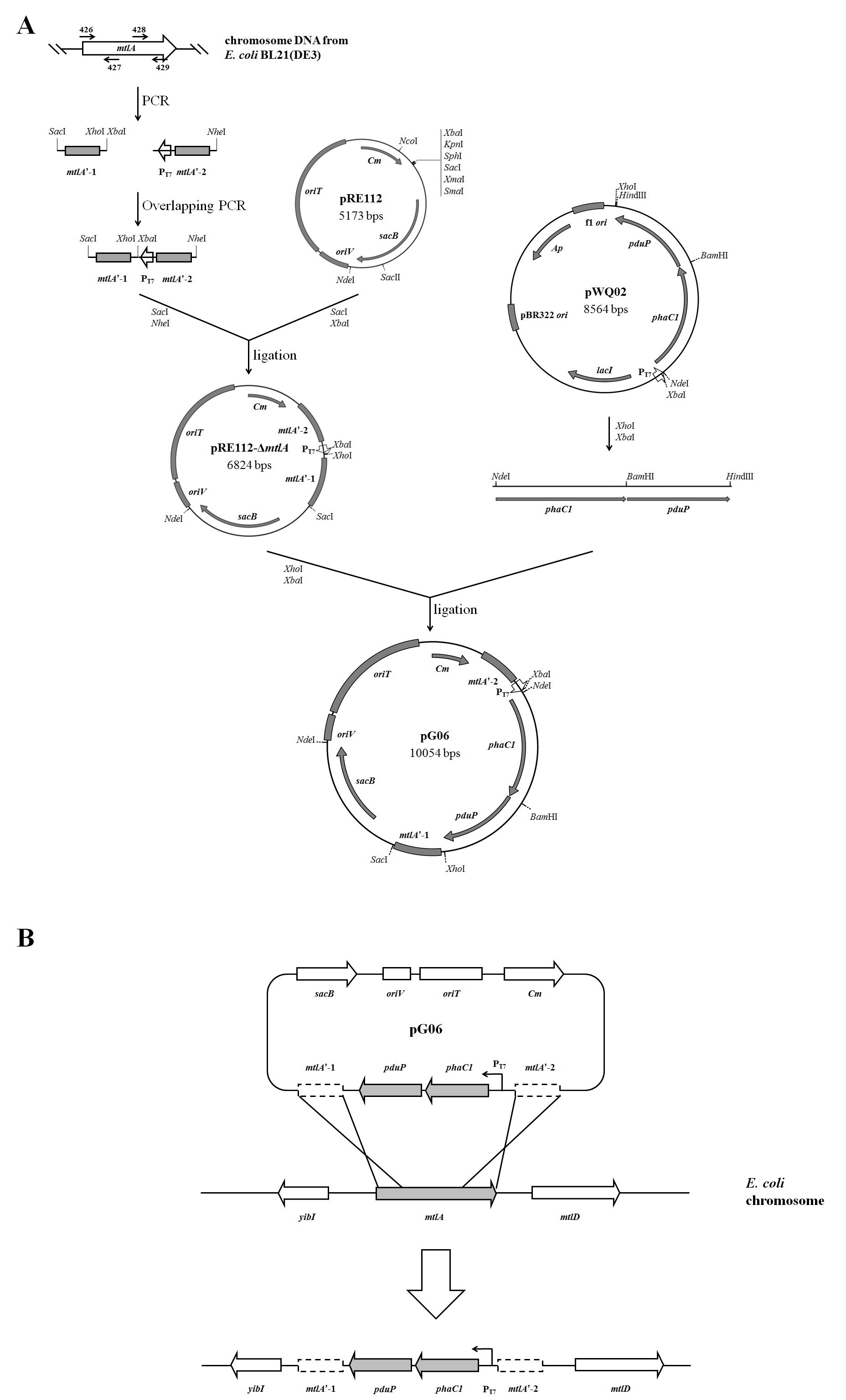


Fig. S3. Construction process of strain carrying *pduP* and *phaC1* genes at chromosomal *mtlA* locus. (A) Construction of suicide vector pG06. (B) Allelic exchange between the suicide vector pG06 and *E. coli* chromosome.
